# Does one size fit all? Differences between islands in Tuvalu and ecological perspectives

**DOI:** 10.7189/jogh.12.03082

**Published:** 2022-12-07

**Authors:** Po-Jen Lin, Tai-Lin I Lee, Chih-Fu Wei, Chih-Wei Shih, Maria Soledad Hershey, Yu-Tien Hsu, Selotia Tausi, Vine Sosene, Pauke P Maani, Malo Tupulaga, Yuan-Hung Lo

**Affiliations:** 1 Taiwan International Cooperation and Development Fund, Taipei, Taiwan; 2 Johns Hopkins University Bloomberg School of Public Health, Baltimore, Maryland, USA; 3 Division of Medical Imaging, Department of Radiology, Far Eastern Memorial Hospital, New Taipei City, Taiwan; 4 Department of Environmental Health, Harvard T.H. Chan School of Public Health, Boston, Massachusetts, USA; 5 Taiwan Technical Mission to Tuvalu, Funafuti, Tuvalu; 6 Department of Social & Behavioural Sciences, Harvard T.H. Chan School of Public Health, Boston, Massachusetts, USA; 7 Tuvalu Department of Agriculture, Funafuti, Tuvalu; 8 Department of Public Health, Tuvalu Ministry of Health, Funafuti, Tuvalu

## UNIQUE CHALLENGES FOR TUVALUANS REGARDING NUTRITION AND FOOD

Tuvalu is a South Pacific Ocean nation consisting of nine atolls (Funafuti, Nanumea, Nanumaga, Niutao, Vaitupu, Nukufetau, Nui, Nukulaelae and Niulakita) and is one of the countries impacted the most by climate change [1]. Due to scarcities of fertile farmland and fresh water, agricultural activities are limited in Tuvalu. Inhabitants often resort to imported produce [2]. These changes have raised concern for food security, particularly during the SARS-CoV-2 lockdown and ongoing climate change [3].

Limited agricultural activities, nutrition strategies, and financing have culminated in an overwhelming obesity rate of 59.9% in adult women and 51.5% in adult men, much higher than the South Pacific regional average of 31.7% and 30.5% for women and men, respectively [4]. Although the prevalence of obesity is primarily a result of individual behaviours, these structural factors, including the consumption of high-calorie imported food, could prompt unhealthy food choices and undesirable health outcomes [5,6].

## EFFORTS IN IMPROVING FOOD SECURITY IN TUVALU

Over the last decade, the Tuvalu Department of Agriculture and the Taiwan International Cooperation and Development Fund have collaborated in building government gardens by introducing compost production facilities, heat-adapted crops, and the use of raised bed gardens. Meanwhile, nutrition courses and recipes using locally sourced ingredients have been developed for public health promotion.

In recent years, the Tuvalu Department of Agriculture has advocated for the concept of a “home garden” (“*fatoaga ite fale*” in Tuvaluan), encouraging Tuvaluans to grow vegetables near their houses, which requires adequate space near the house, care for the crops, and continuous garden maintenance. The attributed benefits of home gardening are well-established in the literature [7,8]. Among Pacific Islands countries, home gardens have the additional potential to promote agricultural diversification and maximize self-sufficiency [9]. Besides increasing the availability of fresh fruits and vegetables, home gardens keep people physically active and decrease stress [7].

## DIFFERENCES BETWEEN THE MAIN ISLAND AND OTHER OUTLYING ISLANDS OBTAINED FROM THE RESULT OF A NATIONWIDE SURVEY

The outlying islanders have traditionally depended on their agroforestry and fishery resources to create sustainable systems which provided for most of their daily necessities. In comparison, Funafuti has a higher urbanization level and relies heavily on imported foods. Understanding differences in farming, dietary consumption, and ecologic conditions can help tailor advocacy campaigns for “home gardens” for each island. To identify factors associated with higher local garden utilization (including home gardens and government gardens) and provide evidence for future policy improvements, we conducted a nationwide survey from February to May 2022 to examine specific demographic determinants. Local interviewers were trained to record subjective demographic data and body measurements such as height, weight, and waist circumference. We assessed local peoples’ home gardens, government gardens use, and other behavioural factors. We approached households in Funafuti, the main island and the eight outlying islands. In each household, interviewers randomly selected one or two adults (>18 years of age) of different genders and conducted face-to-face interviews to collect survey data. The Tuvalu Department of Health approved the study protocol and informed written consent was obtained from each participant. Lastly, regional differences in home garden and government garden utilization and imported/domestic food consumption were estimated using univariate and multivariate logistic regressions.

**Figure:**
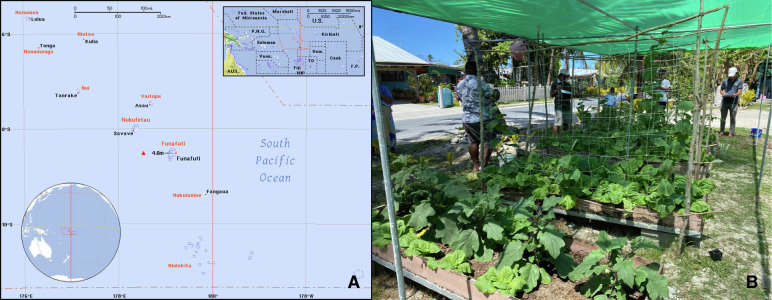
Photo: Panel A: Map of Tuvalu. Source: https://ian.macky.net/pat/map/tv/tv_blu.gif. Permission for use not needed (open-source photo). Panel B: A well-designed home garden in Tuvalu. Source: Yuan-Hung Lo (senior author)’s own collection, used with permission.

A total of 1030 adults (636 main island residents and 394 outlying island residents) were included in the study. Descriptive characteristics showed main island respondents were younger, had higher income, higher education levels, and higher nutrition-related knowledge scores. In contrast, respondents from outlying islands reported more frequent exercise, home gardens use, and government garden visits. We observed no significant differences in gender, body mass index, smoking status, and alcohol consumption between the two groups. Residents of the outlying islands more frequently participated in home gardens and visits to government gardens after adjusting for gender, age, education level, non-communicable diseases (NCDs), income, and smoking status. Other positive predictive factors for government garden visits included older age, higher income, and higher education level, but none were associated with the likelihood of home garden use.

Additionally, Tuvalu’s outlying island residents consumed fewer imported foods. Almost 90% of the main island residents identified rice as their staple food, compared to around 50% in outlying islands whose diet comprised mainly of domestic foods such as swamp taro, taro, or breadfruit. Main island residents consumed chicken more frequently and fish less frequently than outlying islanders. Main islanders purchased more imported vegetables, fruits, sugar-sweetened beverages, and ice cream compared to their counterparts.

## ECOLOGICAL DIFFERENCES

We collected population density and precipitation data for each island. According to the Tuvalu agriculture and fisheries report [10], population density in Funafuti was 2257 persons per square kilometre in 2017, which was much higher than Tuvalu’s average (410 persons per square kilometre) and the eight outlying islands (around 100 to 200 persons per square kilometre). The total population in Tuvalu from 2012 to 2017 decreased by 1.25%; meanwhile, the number of residents in Funafuti increased by 16.3% [11], due to the ongoing migration from the outlying islands to the main island [10]. Overcrowding in Funafuti impacts the already vulnerable ecosystem, limits possible spaces for home gardening, and increases the demand for imported foods. Proper land-use planning has become urgent in Funafuti in order to mitigate the negative consequences of overpopulation.

**Figure 1:**
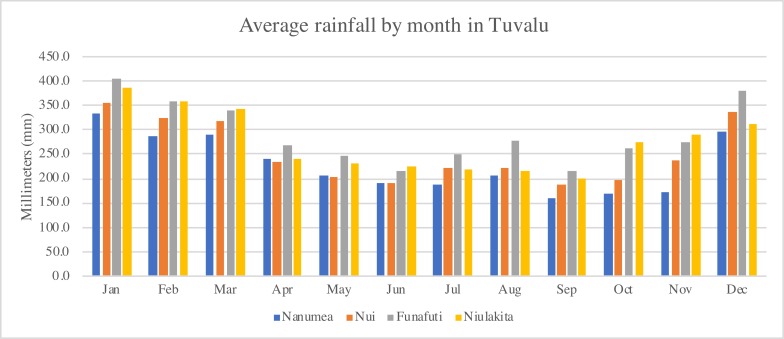
Average rainfall by month in Tuvalu between 1953 and 2022.

An adequate amount of rain is also crucial for agricultural extension. We collected the monthly precipitation data in different islands from the four meteorological stations in Funafuti, Nanumea, Nui, and Niulakita. A 70-year monthly average rainfall from 1953 to 2022 is shown in **Figure 1**.

Nanumea, the northmost outlying island, has the lowest average rainfall among the four islands, except during April and May. Funafuti and Niulakita have relatively more rain compared to the other two islands. Due to geographic proximity, the lack of sufficient water supply during dry seasons in Nanumea, and possibly Nanumaga and Niutao, may pose additional difficulty to home garden promotion. Therefore, the introduction of efficient water storage equipment is necessary for the North outlying islands (Nanumea, Nanumaga and Niutao).

In our survey, we found that residents in the South outlying islands (Niulakita and Nukulaelae) are 4.27 times more likely to use home gardens compared to the Funafuti as the reference group after adjusting for gender, age, education level, NCD diagnosis, income, and smoking status. Meanwhile, the North outlying islands observed an adjusted odds ratio of 1.67, which is a smaller point estimate, but is still significantly higher than the main island. These findings demonstrate land-use heterogeneities between the outlying islands and suggest ecological differences are important when designing effective strategies to promote home gardens on each island.

## TAILORING A SUSTAINABLE FOOD SYSTEM FOR ISLANDS IN TUVALU

This campaign was Tuvalu's first large-scale, nationwide, population-based study on home/government garden utilization and food choices and is among the few studies examining the differences between the main and outlying Tuvalu islands and discussing implications for food system changes in Tuvalu. Only one similar study in the Federated States of Micronesia [12] tested the differences between the main and remote islands. They did not observe any difference in their 98-subject population. Multifaceted approaches should aspire to cultivate sustainable food attitudes and practices in Tuvalu. Ultimately, strategies should empower the local government and residents to maintain long-lasting, self-sufficient food systems against the global obesity pandemic. We engaged local interviewers during the survey process. Through observation and hands-on experiences, the local interviewers experienced direct community contribution and acquired knowledge for future practices.

The adoption of “home gardens” and its associated concepts can reduce the impact of climate change on food security, especially in small island developing states (SIDS). People living in these countries have become highly dependent on nutrient-poor imported food, reflected by their above-global average obesity and NCD rates [13]. Home gardens can provide more affordable and nutrient-dense sources of vegetables and fruits, consequently relieving the negative health outcomes caused by imported foods. Additionally, the farm-to-table model significantly decreases carbon footprints incurred from global shipping, which contributes to about 3% of total anthropogenic carbon dioxide emissions [14]. Our findings revealed that environmental factors determined parts of home garden acceptance and that the endeavour should be tailored to each island's geographic characteristics, including population density and rainfall. This is consistent with a summary article on home gardening around the world by socio-economist Robin Marsh, who concluded that utilizing local materials (including soil and water) is one ingredient to successful home garden promotion. Six other key points include steps to 1) integrate nutrition awareness and education, 2) crop locally adapted varieties, 3) work with experienced households to build on traditional home gardening methods, 4) engage village leaders, 5) involve whole families, especially women, in outcome distribution, and 6) regular monitoring and fine-tuning [15]. This project currently employed all but points 3) and 5) listed above to promote home gardening in Tuvalu. There appeared to be a discrepancy in home garden needs and supplies between the main and outlying islands. Main islanders may benefit more from home garden use as they suffer more from the health consequences of unhealthy imported food, yet the densely populated cities limit available farmlands. Coordination of the supply chain between the main island and outlying islands is warranted. Government gardens, which offer advantages similar to home gardens without the same level of labour requirements, appear to be a feasible solution on the main island. The findings on higher education levels and government garden use support ongoing educational interventions to improve food knowledge.

In conclusion, fundamental differences were observed regarding farming, dietary consumption, and ecologic conditions between the main island and the outlying islands of Tuvalu. This suggests that public education and policies should be tailored to these differences and consider the varying demographics and environments in the main island and other smaller islands in the SIDS, such as Tuvalu [16].
